# Pigmented Villonodular Synovitis Presenting at an Atypical Site: A Case Report

**DOI:** 10.7759/cureus.31452

**Published:** 2022-11-13

**Authors:** Samiksha D Lokhande, Nareshkumar S Dhaniwala, Ashutosh Lohiya, Aditya Joseph Chirayath

**Affiliations:** 1 Orthopaedic Surgery, Jawaharlal Nehru Medical College, Datta Meghe Institute of Medical Sciences, Wardha, IND

**Keywords:** giant cell tumor, reoccurrence, illio-tibial band, atypical location, pigmented villonodular synovitis

## Abstract

Pigmented villonodular synovitis (PVNS) is an idiopathic villous overgrowth and pigmentation of the synovial membrane of a single joint. It is an uncommon condition characterized by yellow or yellowish-brown colour due to deposits of cholesterol and hemosiderin, excessive secretion of yellowish-brown synovial fluid, and the formation of brownish chocolate synovial tissue. This condition commonly occurs at the knee joint at the age of 20-50 years. Here we present a case of a 75-year-old male with PVNS involving the lower third of the right thigh who came to the surgery department and was eventually referred to the orthopedic department on the basis of investigations performed. It is a case of PVNS at a unique location on the lower third of the right thigh. The swelling was painless initially, but the pain increased over a duration of 10 months. Clinically, the mass was suspected to be bursitis or lipoma with features of mild inflammation in the overlying skin. Magnetic resonance imaging (MRI) suggested a swelling of 100*70*40 mm in dimension with the possibility of PVNS. Ultrasonography (USG) of the mass and fine needle aspiration cytology (FNAC) supported the diagnosis of PVNS. An excisional biopsy of the swelling was submitted. Per-operatively, there were typical features of PVNS. The swelling was situated superficial to the iliotibial band. There was no defect or gap in the iliotibial tract, and the swelling didn’t have any continuity to the knee joint. The occurrence of synovial tissue without any attachment to the joint or tendon is rare and hence reported.

## Introduction

Pigmented villonodular synovitis (PVNS) is classified as a tenosynovial giant cell tumor (TGCT) [1]. PVNS affects the soft tissue of the joints, ligaments, and the associated muscles [2]. The most typical locations in the body affected are the knees, hips, and ankles and the commonest is the knee. The disease slowly starts and its symptoms gradually progress [1]. PVNS has aggressive proliferative elements present in it. PVNS is more aggressive than giant cell tumors of the tendon sheath (GCT-TS). PVNS cases have a defect in the series of chromosomes [3]. This chromosome translocation leads to an increase in the colony-stimulating factor 1 (CSF1) [3]. The increase in such factors causes enlargement of the soft tissues, hypertrophy, and hyperplasia of the synovial membrane present over the joints.

The disease diagnosis is delayed due to gradual progressive symptoms and may lead to secondary osteoarthritis, instability, and gross erosive changes in the bones of the joint. In both males and females, the ratio of PVNS is the same. Still, some studies prove that females are more affected by this tumor [4]. Age indicates that PVNS does not affect infants and children [5]. In the early stages of PVNS, the patient has idiopathic edema over the diseased joint [6]. Over time, the symptoms like pain and edema gradually increase, restricting the movement of joints. In further stages, the condition causes excessive secretion of brownish synovial fluid in the joint, which leads to the accumulation of coagulating factors and other components of blood causing joint restriction, and the patient is unable to move the joint [7]. The patient in this stage may have difficulty doing simple movements like flexion, extension, abduction, and adduction. It is seen mainly in the single joint, but multiple joints can also be affected tumors can also be seen in this disease [5]. The primary location of the disease is the large joints of the limbs [6].

## Case presentation

A 75-year-old male patient, farmer by occupation and resident of a rural area, was admitted to the surgery department in our tertiary care centre in central India. After a detailed examination, investigations, and orthopaedic reference, he was transferred to the orthopedic department. The patient presented to the hospital with the chief complaint of swelling on the right lower thigh for 10 months. The swelling had appeared spontaneously without any trauma, fever, or swelling in the knee region. It was small to begin with and slowly increased in size without pain or difficulty in knee movement or ambulation. It grew to the size of a prominent lemon and started giving mild discomfort to the patient. It never regressed on its own, and the patient wanted it to be treated due to pain and cosmetic reasoning. There was no history of fever, cough, breathlessness, nausea, and vomiting. The patient had no history of diabetes mellitus, tuberculosis, bronchial asthma, hypertension, or knee osteoarthritis. Family history was not significant. The patient belonged to a lower socioeconomic status. He had consulted a local practitioner for the swelling. He was given tablet cefixime 200mg 12 hours for five days and tablet aceclofenac 100 mg 12 hourly for five days, but they did not result in any change in the swelling size or reduction in the pain. He had not undergone any surgical intervention for the present complaint or other diseases.

**Figure 1:**
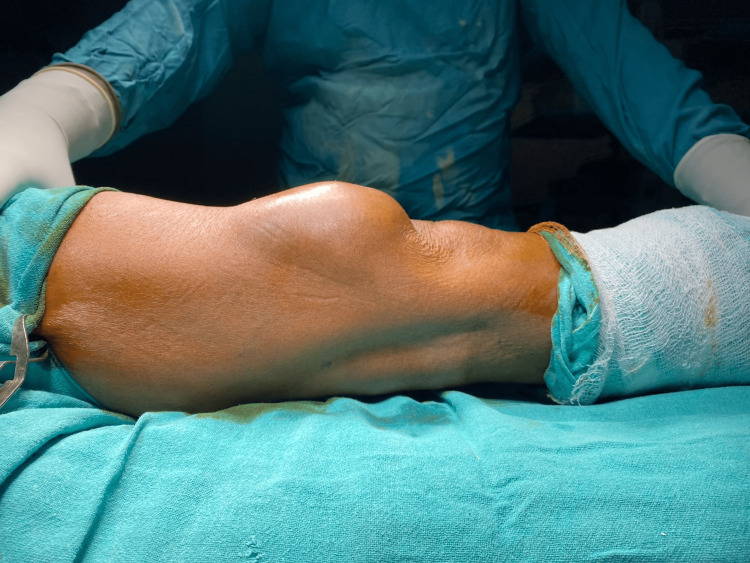
Swelling on the anterolateral aspect of the lower third of the thigh

The patient was 165 cm tall, weighing 71 kg, and had a BMI of 26.08. His pulse was 78 beats per minute, respiratory rate 16 per minute, and blood pressure 130/90 mmHg. His systemic examination showed no abnormalities in the cardiovascular system, respiratory system, gastrointestinal tract, or central nervous system. Local examination revealed a single swelling of 5×7x4 cm on the anterolateral aspect of the lower third of the right thigh, as shown in Figure 1.

There was a mild rise in local temperature. The surface of the swelling was smooth, the margins were well-defined, and the consistency was firm/cystic. The swelling was mobile freely in the transverse plane and was not attached to the underlying bone or muscles. The overlying skin was free and pinchable. The transillumination test was positive. There was no thickening of synovial tissue appreciable in the suprapatellar, parapatellar, or infrapatellar region. There was no effusion present. The knee joint line was mildly tender medially, and the knee had a painless range of movement from 0 degrees to 130 degrees. Differential diagnoses considered were bursitis, lipoma, or organised abscess.

The patient was investigated and the following investigations were done: complete blood count (CBC), peripheral smear, coagulation profile, kidney function test (KFT), liver function test (LFT), random blood glucose (RBS), and X-Ray of the knee, USG of the swelling, cytology of the aspirate from the swelling (fine needle aspiration cytology [FNAC]), and MRI. The findings of the investigations carried out are given in Table 1.

**Table 1:** showing Investigations performed CBC: Complete Blood Count; Hb: Haemoglobin; MCHC: Mean Corpuscular Haemoglobin Count; MCV: Mean Corpuscular Value; MCH: Mean Corpuscular Haemoglobin; RBC: Red Blood Cell; WBC: White Blood Cells; HCT: Hematocrit; RDW: Red Cell Distribution; APTT: Activated Partial Thromboplastin Time; INR: International Normalized Ratio; KFT: Kidney Function Test; LFT: Liver Function Test; ALT: Alkaline Aminotransferase Test; AST: Aspartate Aminotransferase Test; SGOT: Serum Glutamic-Oxaloacetic Transaminase; SGPT: Serum Glutamic Pyruvic Transferase; BC: Conjugated Bilirubin; BU: Unconjugated Bilirubin; RBS: Random Blood Sugar; ZN Staining: Ziehl-Neelsen Staining; HbSAg: Hepatitis B; HCV: Hepatitis C.

Sr. No.	Date	Name of the test	Result	Reference Range
1	09/09/2022	CBC		
1.1		Hb%	11.4 g/dl	13-17
1.2		MCHC	33.3 g/dl	31.5-34.5
1.3		MCV	89.4 fl	83-101
1.4		MCH	29 pq	27-32
1.5		Total RBC count	3.63 X 10^6 ^/ μL	4.5-5.5
1.6		Total WBC count	9700 X 10^3^ / μL	4.0-10.0
1.7		HCT	34.3 %	40-50
1.8		RDW	13.3 %	11.6-14
1.9		Total Platelet count	2.26 %	150-400
1.1		Monocytes	3 %	0-10
1.11		Granulocytes	75 %	50-70
1.12		Lymhocytes	70 %	20-40
1.13		Eosinophills	2 %	0.0-6.0
1.14		Basophill	0 %	<2
2	10/09/2022	Coagulation profile		
2.1		APTT-Control	29.5 seconds	30-40
2.2		APTT-Patient	30.6 seconds	30-40
2.3		Prothrombin time-Control	11.9 seconds	11-13.5
2.4		Prothrombin time-Patient	12 seconds	11-13.5
2.5		INR	1	<1.1
3	10/09/2022	Urine		
3.1		Urine albumin	Nil	
3.2		Urine sugar	Nil	
3.3		Crystals		
3.4		Epithelial cells	1.2 CELLS/HPF	1.0-5.0
3.5		Pus cells	1.2 CELLS/HPF	0-5
3.6		RBC	1.2 CELLS/HPF	<4
4	10/09/2022	Peripheral smear	RBC-Normocytic Normochromic Platelets-Adequate on smear. No Haemoparasite seen.	
5	10/09/2022	KFT		
5.1		Urea	48 mg/dl	adult: 17-43
5.2		Creatine	2 mg/dl	0.6-1.1
5.3		Sodium	145 mEq/L	135-145
5.4		Potassim	4.5 mmol/L	3.6-5.2
6	10/09/2022	LFT		
6.1		Alkaline Phosphate	77 U/L	45-129
6.2		ALT(SGPT)	25 U/L	<45
6.3		AST(SGOT)	30 U/L	<35
6.4		Total Protein	7 g/dl	5.7-8.2
6.5		Albumin	4 g/dl	3.2-4.8
6.6		Total Bilirubin	0.5 mg/dl	0.3-1.2
6.7		BC	0.2 md/dl	<0.3
6.8		BU	0.3 mg/dl	0-0.9
6.9		Globulin	3 g/dl	2.50-3.40
7	10/09/2022	RBS-Glucose-Plasma Random	RBS-Glucose-Plasma Random	
8	10/09/2022	Micro Report		
8.1		Culture	No Growth	
8.2		Gram Staining	Plenty of pus cells were seen. No organism seen	
8.3		ZN Staining	Negative for acid-fast bacilli	
9	10/10/2022	Virology		
9.1		HbSAg_Reactive	Non-Reactive	
9.2		HCV_Reactive	Non-Reactive	
9.3		Other_Name	HIV	

**Figure 2:**
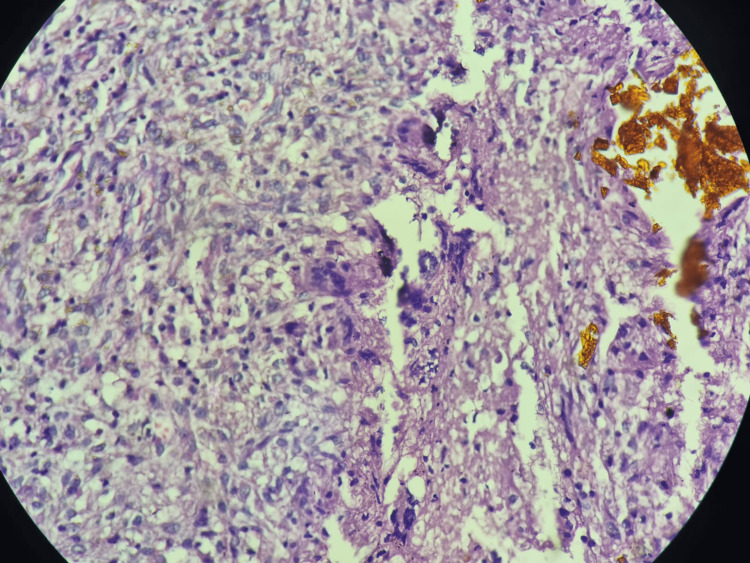
Histopathology

X-Ray knee showed reduced joint space more on the medial aspect and osteophytes at the non-weight bearing area. The case diagnosis was suspected based on USG, which showed a homogenously hypoechoic lesion with few interceptions located over the anterior aspect of the right lower thigh in the subcutaneous plane and moderate peripheral vascularity over the lesion wall. MRI of the right thigh with knee showed 100*70*40mm collection in subcutaneous tissue located 50mm above the knee joint. Histopathology of the excised specimen showed a yellowish-orange cystic area on the cut section. Microscopic examination of the given tissue showed histopathological features suggestive of PVNS as shown in Figure 2. There were lymph nodes that showed characteristics of follicular hyperplasia and sinus histiocytosis. FNAC showed cytomorphology suggesting PVNS without malignant cells.

**Figure 3:**
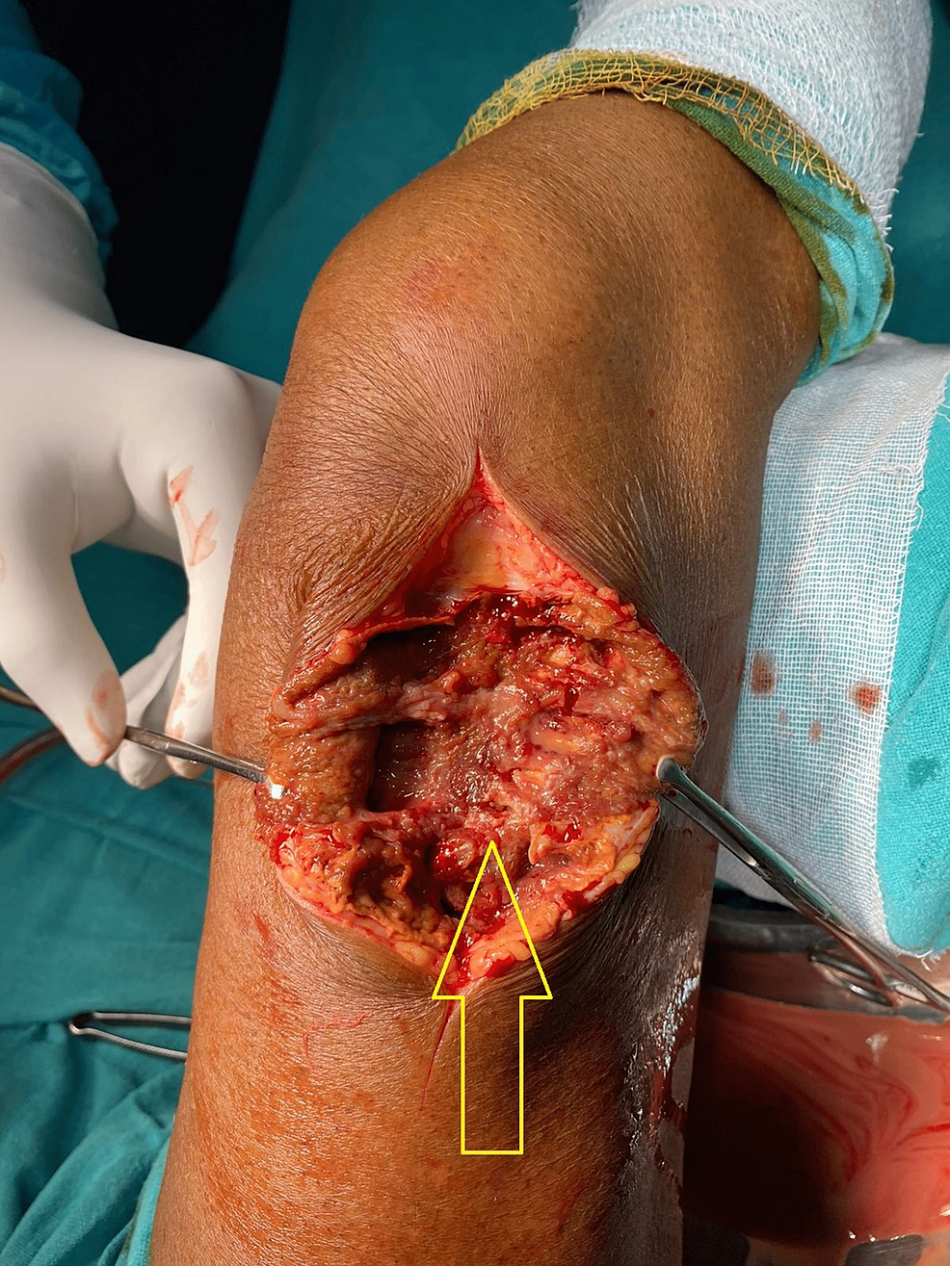
Yellowish-brown swelling typical of pigmented villonodular synovitis

**Figure 4:**
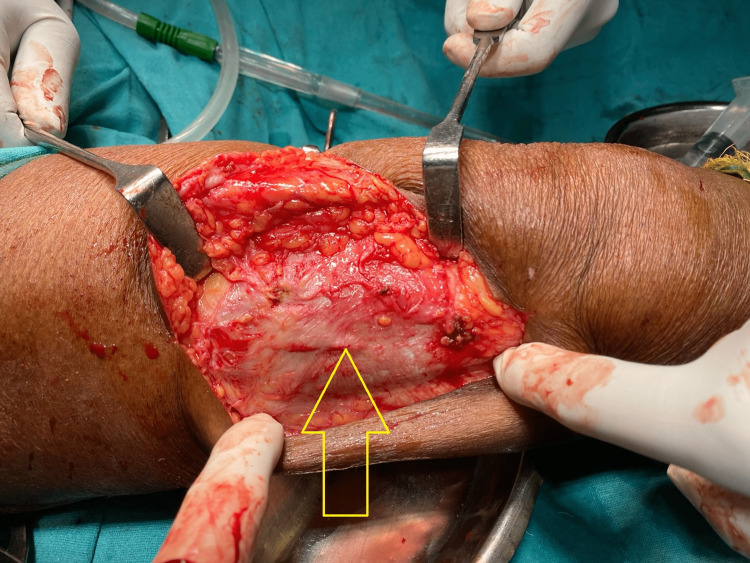
Ilio-tibial band without any defect after excision of swelling

The diagnosis was confirmed by FNAC and supported by a preoperative picture of PVNS at the time of excisional biopsy. There was necrotic brownish tissue present superficially. The swelling consisted of chocolate brownish synovial plicae typical of PVNS, as shown in Figure 3. The swelling was lying superficial to the iliotibial tract, and there was no attachment or continuity with the knee joint, as shown in Figure 4.

The prognosis of PVNS is supposed to be aggressive, and its recurrence is known. An excisional biopsy of the swelling and material was submitted for culture, sensitivity, and histopathology (Figure 4).

**Figure 5:**
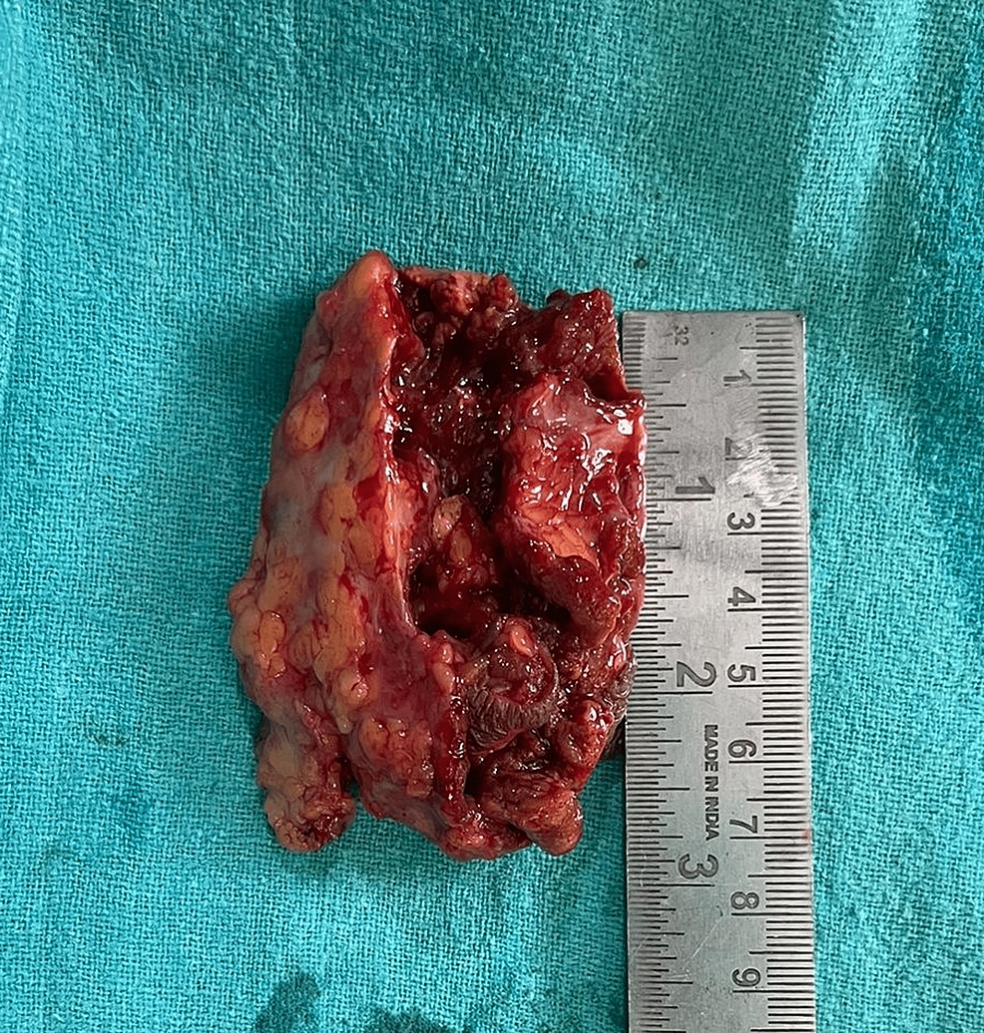
Excised swelling of size 25*30mm

Postoperatively, the patient was kept nil by mouth for six hours with intravenous (IV) fluids, including 2 units of normal saline at 100 ml/hr and 2 units of ringer lactate at 100 ml/hr dose. For pain control, an Injection of paracetamol 2 ml in 100 ml normal saline was given. After destarvation, the patient was given a prophylactic antibiotic injection of ceftriaxone 1 g twice daily for three days, and a supportive drug of vitamins and calcium was prescribed as a tab. Calcium 500 mg once daily, tablet Limcee 500 mg twice daily and tablet paracetamol 650 mg thrice a day. On the fourth postoperative day, the dressing of the patient was changed. There was no discharge and the wound healed uneventfully. His stitches were removed on the 12th postoperative day.

The patient was under follow-up. He had a pre-surgery range of knee movement and does his routine activity without interruption. The patient was advised to contact the concerned physician for the reoccurrence of the swelling. After administration of the drug, the patient had no side effects or symptoms in the postoperative period. There were no adverse and unanticipated events.

## Discussion

PVNS has been described to be of two types. The standard type involves the synovium of the joint diffusely and leads to gradually progressive changes in the joints causing recurrent hemarthrosis and secondary osteoarthritis. The other type is presented as localized swelling in the synovium. Most often, PVNS presents with pain and a limited range of movement in the diffuse form [8]. Despite being a benign condition, recurrence rate after initial resection has been reported to be between 8% to 60%. The localized type of PVNS occurs in the third to fifth decade with very late symptoms [9].

A thick orange-brown fluid on aspiration containing cholesterol in large amounts is pathognomonic of this condition. Surgical excision is the treatment of choice [8]. It can be done by open subtotal synovectomy or arthroscopic synovectomy. Radiation therapy in case of massive swelling due to diffuse PVNS can be used to shrink the swelling [8].

The reported case is a localized type of PVNS which has presented primarily due to swelling and mild discomfort. Aspiration showed the classical orange-brownish fluid. USG and MRI of the knee showed features suggestive of mass in the subcutaneous location containing tissue and fluid. FNAC, per-operative picture, and histopathology all showed the features typical of PVNS. The swelling was not in continuity with the knee and was lying superficial to the iliotibial band. As bursae and tendon sheaths are related to synovium in their origin, xanthomatous growth such as PVNS may develop in these also [9]. The iliotibial band is like a tendon in its structure; hence PVNS might have developed at this site. There is no mention in the literature regarding PVNS at an atypical location. Several authors have pointed out a link between an old joint injury and the development of PVNS, but it has not been demonstrated across other studies [10]. Metastases of PVNS, both malignant and benign, are rare, but metastasis to the lung, muscles, and lymph node has been reported.

## Conclusions

PVNS is a condition that usually affects the joints of the body, which mainly includes the knee joint and hip joint. According to this case, PVNS is reported above the knee joint at the lower third of the thigh. This might be due to the presence of synovial tissue present at the site of PVNS. The patient was operated on by excision of the mass with a good outcome.
